# Active Tracking-based cardiac triggering for MR-thermometry during radiofrequency ablation therapy in the left ventricle

**DOI:** 10.3389/fcvm.2022.971869

**Published:** 2022-08-25

**Authors:** Ronald Mooiweer, Rainer Schneider, Axel Joachim Krafft, Katy Empanger, Jason Stroup, Alexander Paul Neofytou, Rahul K. Mukherjee, Steven E. Williams, Tom Lloyd, Mark O'Neill, Reza Razavi, Tobias Schaeffter, Radhouene Neji, Sébastien Roujol

**Affiliations:** ^1^School of Biomedical Engineering and Imaging Sciences, Faculty of Life Sciences and Medicine, King's College London, London, United Kingdom; ^2^MR Research Collaborations, Siemens Healthcare Limited, Camberley, United Kingdom; ^3^Siemens Healthcare GmbH, Erlangen, Germany; ^4^Imricor Medical Systems, Burnsville, MN, United States; ^5^Centre for Cardiovascular Science, The University of Edinburgh, Edinburgh, United Kingdom; ^6^Physikalisch-Technische Bundesanstalt (PTB), Braunschweig, Germany

**Keywords:** active tracking, MR-thermometry, cardiac triggering, RF ablation, ECG

## Abstract

Cardiac MR thermometry shows promise for real-time guidance of radiofrequency ablation of cardiac arrhythmias. This technique uses ECG triggering, which can be unreliable in this situation. A prospective cardiac triggering method was developed for MR thermometry using the active tracking (AT) signal measured from catheter microcoils. In the proposed AT-based cardiac triggering (AT-trig) sequence, AT modules were repeatedly acquired to measure the catheter motion until a cardiac trigger was identified to start cardiac MR thermometry using single-shot echo-planar imaging. The AT signal was bandpass filtered to extract the motion induced by the beating heart, and cardiac triggers were defined as the extremum (peak or valley) of the filtered AT signal. AT-trig was evaluated in a beating heart phantom and *in vivo* in the left ventricle of a swine during temperature stability experiments (6 locations) and during one ablation. Stability was defined as the standard deviation over time. In the phantom, AT-trig enabled triggering of MR thermometry and resulted in higher temperature stability than an untriggered sequence. In all *in vivo* experiments, AT-trig intervals matched ECG-derived RR intervals. Mis-triggers were observed in 1/12 AT-trig stability experiments. Comparable stability of MR thermometry was achieved using peak AT-trig (1.0 ± 0.4°C), valley AT-trig (1.1 ± 0.5°C), and ECG triggering (0.9 ± 0.4°C). These experiments show that continuously acquired AT signal for prospective cardiac triggering is feasible. MR thermometry with AT-trig leads to comparable temperature stability as with conventional ECG triggering. AT-trig could serve as an alternative cardiac triggering strategy in situations where ECG triggering is not effective.

## Introduction

Radiofrequency catheter ablation for treating ventricular tachycardia is commonly performed under fluoroscopic and electro-anatomic mapping guidance (1), but remains associated with a relatively high rate of recurrence (up to 50%) (2). The outcome of these procedures is potentially limited by the inability of these guidance systems to accurately characterize the extent of ablation lesions. Indirect parameters such as radiofrequency power/duration and catheter tip temperature/contact force/impendence are monitored but have limited predictive values of permanent ablation lesion extent (3, 4). Furthermore, X-rays can be harmful for both patients and clinical staff.

Magnetic resonance imaging (MRI) guidance is a promising alternative as it enables excellent soft tissue visualization, 3D myocardial scar quantification for arrhythmia substrate characterization, and procedure planning, as well as ablation lesion imaging (5, 6). The latter can be performed during ablation using real-time magnetic resonance (MR) thermometry/dosimetry (7–12) or post-ablation using a variety of sequences (13, 14). MR thermometry, commonly performed using the proton resonance frequency shift technique (15), enables real time pixel-wise assessment of temperature, deep in tissue. Permanent tissue destruction can be predicted using the concept of thermal dose (MR dosimetry), which is based on temperature elevation and time of exposure (16). MR thermometry/dosimetry thus has the potential to enable real-time adjustment of ablation parameters (such as power/duration) to achieve the desired lesion as well as to identify insufficient heating leading to incomplete ablations or excessive heating compromising the safety of the patient.

Cardiac MR thermometry is generally performed using a dynamic multislice single-shot echo-planar imaging (EPI) sequence (~3–5 slices per heartbeat (8–11, 17, 18)). Electrocardiogram (ECG) triggering is employed to ensure the consistency of the cardiac phase across the time series, which is required for accurate temperature and thermal dose mapping. However, in this context, ECG triggering can be unreliable due to the additional [radiofrequency (RF)] hardware and the rapidly switching gradients associated with EPI that are used for fast thermometry acquisitions in moving organs necessary to avoid motion blurring artifacts (10, 17, 19).

MR-compatible ablation catheters often contain active tracking (AT) microcoils to facilitate navigation by tracking the catheter tip in real time using a short-pulse sequence module with low flip angle excitation (10, 11, 20). In this study, we propose a novel prospective cardiac triggering approach for MR thermometry by continuously measuring the catheter position with AT within the MR thermometry sequence and using this signal to compute a surrogate of the cardiac motion. In this proof-of-concept study, the proposed active tracking-based triggering (AT-trig) MR thermometry sequence is evaluated in a beating heart phantom and compared to conventional ECG-triggered MR thermometry *in vivo*.

## Materials and methods

### Proposed AT-triggered MR thermometry sequence and real-time AT signal processing

**Figure 1:**
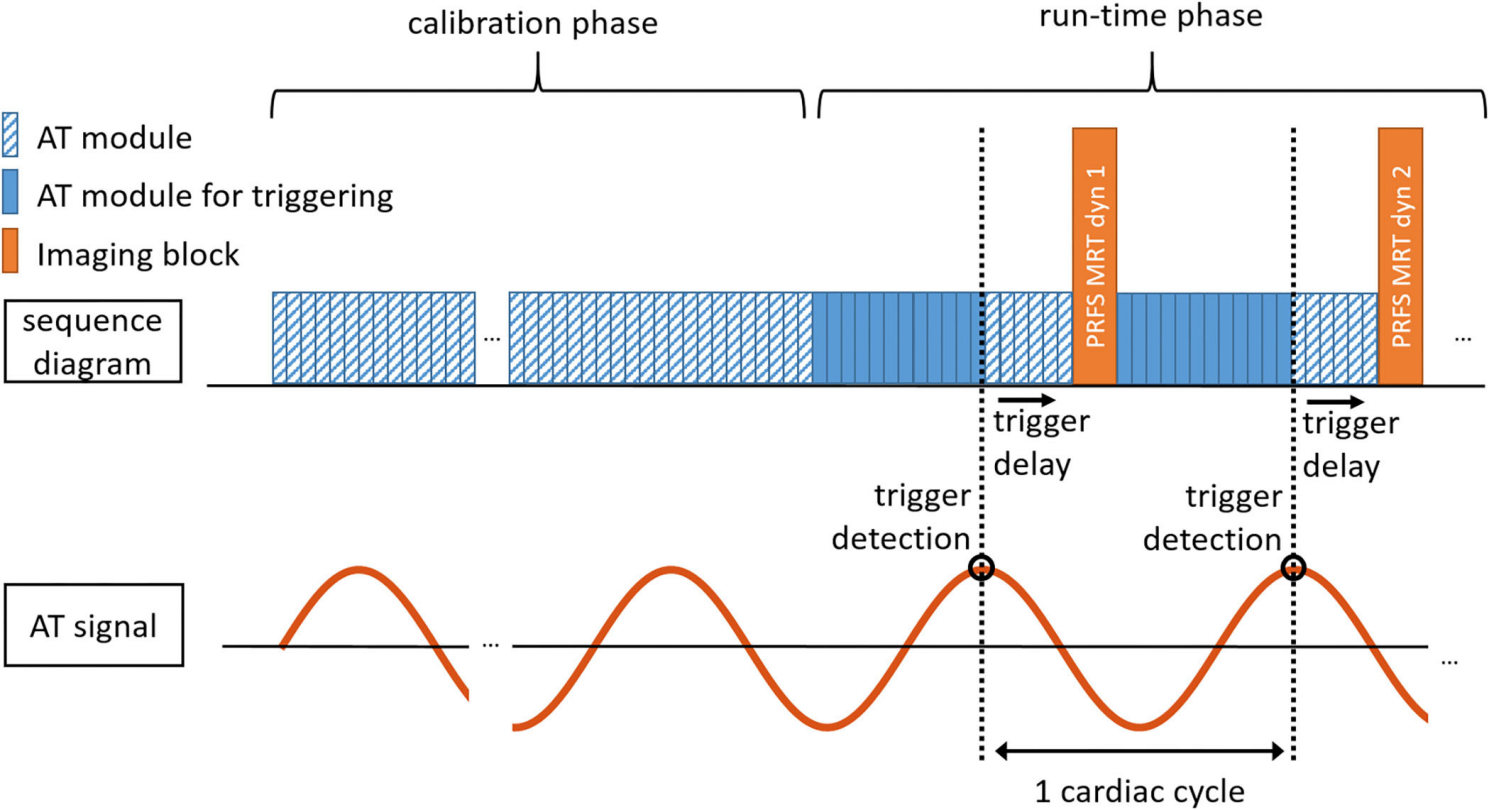
Schematic diagram of AT-triggering-based MR thermometry. After a calibration phase to characterize the AT signal (duration: 16 s), the sequence automatically proceeds to the run-time phase in which MR thermometry is performed in synchronicity with the cardiac cycle through repeated measurements of the AT signal. During each imaging block (dynamic image number 1, 2, etc.), all prescribed slices are acquired following a shared trigger. PRFS, Proton resonance frequency shift; MRT, MR thermometry; dyn, dynamic image number; AT module for triggering, AT module where trigger detection is performed.

The pulse diagram of the proposed AT-trig MR thermometry sequence is shown in Figure 1. The sequence consists of an AT signal calibration phase (16 s) followed by a run-time phase. During the calibration phase, the catheter displacement pattern is continuously monitored using uninterrupted AT module acquisitions. The cardiac motion component of the catheter displacement is extracted from the AT signal and used to determine a cardiac trigger criterion. During the run-time phase, for each cardiac cycle, AT modules are continuously repeated until a trigger event is recorded that initiates the start of each dynamic MR thermometry imaging block in which all prescribed slices are acquired using multislice single-shot EPI.

#### AT signal generation

An MR-compatible RF ablation catheter with two AT microcoils located near the catheter tip (Vision-MR Ablation Catheter, Imricor Medical Systems, Burnsville, MN) was used in this study. The catheter was connected to the receive unit of the MR scanner to enable real-time processing of the AT data as a part of the reconstruction pipeline. The AT pulse sequence module measured the microcoils position using projection imaging (20): for each spatial dimension, a nonselective RF excitation pulse was followed by a one-dimensional gradient readout during which the spatially encoded signal was received by the AT microcoils. This was repeated for each of the 3 spatial dimensions and dephasing gradients were used to increase SNR (21). The total AT module length was 25 ms, in which the 3D position of the two AT microcoils could be determined. In conventional applications of AT for continuous 3D catheter visualization, both microcoils are used to determine the position and orientation of the catheter tip. In this work, the AT signal is used to obtain a surrogate of the cardiac motion during RF ablation by exploiting the periodicity of the catheter displacement induced by physiological motion. The periodicity of the coils' displacement due to physiological motion is expected to be the same for both coils; therefore, the signal from only 1 of the 2 microcoils was used. The catheter motion experienced as result of cardiac contractions is truly 3D and depends on the orientation of the heart and the particular position of the catheter in the LV. To reduce the dimensionality of this motion trace and facilitate its processing, the AT signal was defined as the L2 norm of the coil's coordinate vector.

#### AT signal filtering

First, outliers were rejected to remove occasional incoherent measurements from the measured AT signal. Outliers were defined as exceeding the range of motion by two times the central 90% motion range of the AT signal acquired during the calibration phase. The AT signal was then resampled to a uniform temporal resolution of 40 Hz, matching the AT module length of 25 ms. A bandpass filter was applied in real time to remove low and high frequencies associated with respiratory motion and noise, respectively (22). The bandpass filter was a finite impulse response filter, designed using the *designfilt* function in MATLAB (v2018a, The MathWorks, Nantucket, MI) with a minimum frequency of 40 beats per minute (BPM, 0.67 Hz), a maximum frequency of 150 BPM (2.5 Hz), and a filter order of 400 (i.e., the filter was applied to 10 s of resampled AT signal). The most recent AT position after filtering was kept as the filtered AT signal.

#### Calibration phase

The aim of the calibration phase is to define an absolute AT signal threshold (*S*_*Thres*_) that can be used for triggering on the periodic global extrema of the AT signal while avoiding triggering on local extrema. To this end, AT modules were acquired continuously during the calibration phase and filtering as described in the previous section was applied after 11 s. After having continuously collected 5 s of filtered AT signal, covering several cardiac cycles, *S*_*Thres*_ was defined as the median of the filtered AT signal position. After this calibration phase, 16 s in total, the sequence automatically proceeded to the run-time phase.

#### Run-time phase

During the run-time phase, AT modules were continuously acquired until a cardiac trigger event was detected. Trigger detection was performed after each new AT signal measurement and corresponding filtering, as described in Section AT signal filtering. A trigger event was defined when all of the following 3 conditions were met:

1. The last 3 filtered AT signal entries contained an extremum (peak or valley) at the middle sample. Switching between peak or valley detection was implemented to help in synchronizing the thermometry acquisition with the desired cardiac phase (systole/diastole).
2. The last 3 AT positions were measured sequentially, to avoid triggering on a set of AT signals that were measured with a large time gap in between them, for example, before and after thermometry imaging.
3. The filtered AT position of the suggested trigger point was superior (peak triggering) or inferior (valley triggering) to *S*_*Thres*_.

Upon trigger detection during the reconstruction process, a trigger event is sent immediately to the measurement system that initiates the thermometry imaging block after a user-defined trigger delay to enable imaging in the desired cardiac phase. The trigger delay time was filled with additional AT modules to minimize the gap in continuous AT signal for filtering, and trigger detection was disabled for these additional modules.

### Experimental validation

The proposed sequence was evaluated in a beating heart phantom experiment as well as *in vivo* in a porcine animal model. All experiments were performed on 1.5T scanners (MAGNETOM Aera, Siemens Healthcare, Erlangen, Germany). All animal experiments were approved by the American Preclinical Services (APS) Institutional Animal Care and Use Committee (IACUC).

#### Beating heart phantom experiment

The purpose of the phantom experiment was to assess whether: 1) the AT signal was precise enough to serve as basis for cardiac triggering, 2) the scanner-implemented AT-trig feedback mechanism performed fast enough to serve as cardiac trigger in an MR thermometry sequence before moving on to *in vivo* experiments. A beating heart phantom (DHP-MRI, Shelley Medical Systems, London, ON) was used to mimic contractile motion of the ventricles at 60 BPM. Since there was no respiratory motion in this phantom experiment, AT-trig did not use signal filtering and no AT modules were added during the trigger delay. A simplified first trigger condition was used: the signal was required to be on a positive slope instead of at an extremum. The trigger delay was set to acquire MR thermometry images in the compressed (systolic) state of the phantom.

To compare AT-trig thermometry to best-case thermometry (in absence of an ECG signal), dynamic thermometry imaging was also performed with the phantom statically positioned in the compressed phase (hereafter referred to as “motion-free”). An imaging interval of 1 s was used to achieve MR signal similar to a sequence triggered at 60 BPM. To mimic a situation where cardiac triggering is absent or faulty, MR thermometry imaging was performed with the phantom in motion (60 BPM) and an imaging interval of 80 BPM (hereafter referred to as “untriggered”).

In all experiments, MR thermometry was obtained using the proton resonance frequency shift method (23), acquired using single shot EPI. Other parameters were as follows: dynamics = 60, slices: 1, TR = 70 ms, TE = 16 ms, α = 60°, FOV = 240 x 240 mm^2^, voxel size = 2.1 x 2.1 mm^2^, slice thickness = 5 mm, GRAPPA = 2, partial Fourier = 0.75, bandwidth 1,860 Hz/pixel. AT parameters were as follows: α = 7°, trigger delay: 500 ms. Temperature change maps were calculated using the first image of the series as reference phase image, and temperature stability was defined as the standard deviation over time per voxel. Voxels inside a myocardial mask based on the motion-free images were grouped to calculate mean and standard deviation values to compare the 3 methods.

#### *In vivo* MR thermometry stability study

To evaluate AT-trig *in vivo*, MR thermometry was performed in a porcine animal model under general anesthesia with mechanical ventilation. The catheter was positioned sequentially at six different locations within the LV. At each position, three dynamic MR thermometry series were acquired: 1) using conventional ECG triggering, 2) using AT-trig with peak triggering, and 3) using AT-trig with valley triggering. The heart rate immediately following AT-trig experiments was determined from the ECG signal to compare this expected RR interval to the time interval between successive AT-based triggers.

All MR thermometry measurements used a single-shot EPI sequence with the following acquisitions parameters: dynamics = 150, slices = 3, TR = 93 ms, TE = 17 ms, α = 60°, FOV = 180 x 180 mm^2^, voxel size = 1.6 x 1.6 mm^2^, slice thickness = 5 mm, GRAPPA = 2, partial Fourier = 0.75, bandwidth 1,540 Hz/pixel. The position of the imaging slices was adjusted to the catheter tip position for each of the six locations. Saturation slabs were placed perpendicular to the short axis slices to allow a reduced field of view imaging, and in parallel to the slices to suppress blood signal. AT parameters were as follows: α = 7°, trigger delay = 150 ms. The AT coil that showed the highest signal level in a separate continuous AT acquisition (data not shown) was chosen for the entire experiment.

Temperature map reconstruction was achieved using the following steps, as previously described (11). To remove respiratory-induced phase variations, a look-up-table was first created from the first 40 dynamics containing co-registered phase images. For image registration, the first dynamic was used as a reference image, and a non-rigid motion field between each phase image and the reference phase image was estimated using an optical flow algorithm. The motion field was applied on the complex data to prevent artifacts due to phase wraps. Co-registered phase images were then generated from the registered complex data. After look-up table creation, temperature maps were generated for each dynamic as follows. Each new phase image was registered to the reference position as described above. The most similar co-registered phase image from the look-up table was then selected and used together with the current registered phase for temperature estimation using the proton resonance frequency shift technique. To reduce the influence of noise, a finite impulse-response filter was finally applied to the temperature maps across the temporal dimension. A GPU implementation of the optical flow algorithm was used as previously described to demonstrate the real-time capability of the reconstruction process (24).

Pixel-wise stability of thermometry was computed as the temperature standard deviation across the 110 remaining dynamics and was assessed for each triggering method and position. The analysis was performed for all pixels inside the myocardium and all slices. Areas inside the myocardium containing a signal void induced by the susceptibility of the catheter components were excluded from the analysis. Stability data measured in the myocardium of the 3 slices were pooled together to determine the mean and standard deviation of the stability per triggering method and experimental location.

#### Feasibility study during *in vivo* RF ablation

The *in vivo* feasibility of the proposed approach during RF ablation is demonstrated in one location (position 6 of the stability study). For the ablation experiments, the same AT-trig sequence with valley triggering, as used for the stability study, was employed. RF ablation was started after 40 dynamics of MR thermometry. The RF ablation power was 30 W and had a duration of 30 s.

## Results

### Beating heart phantom experiment

**Figure 2:**
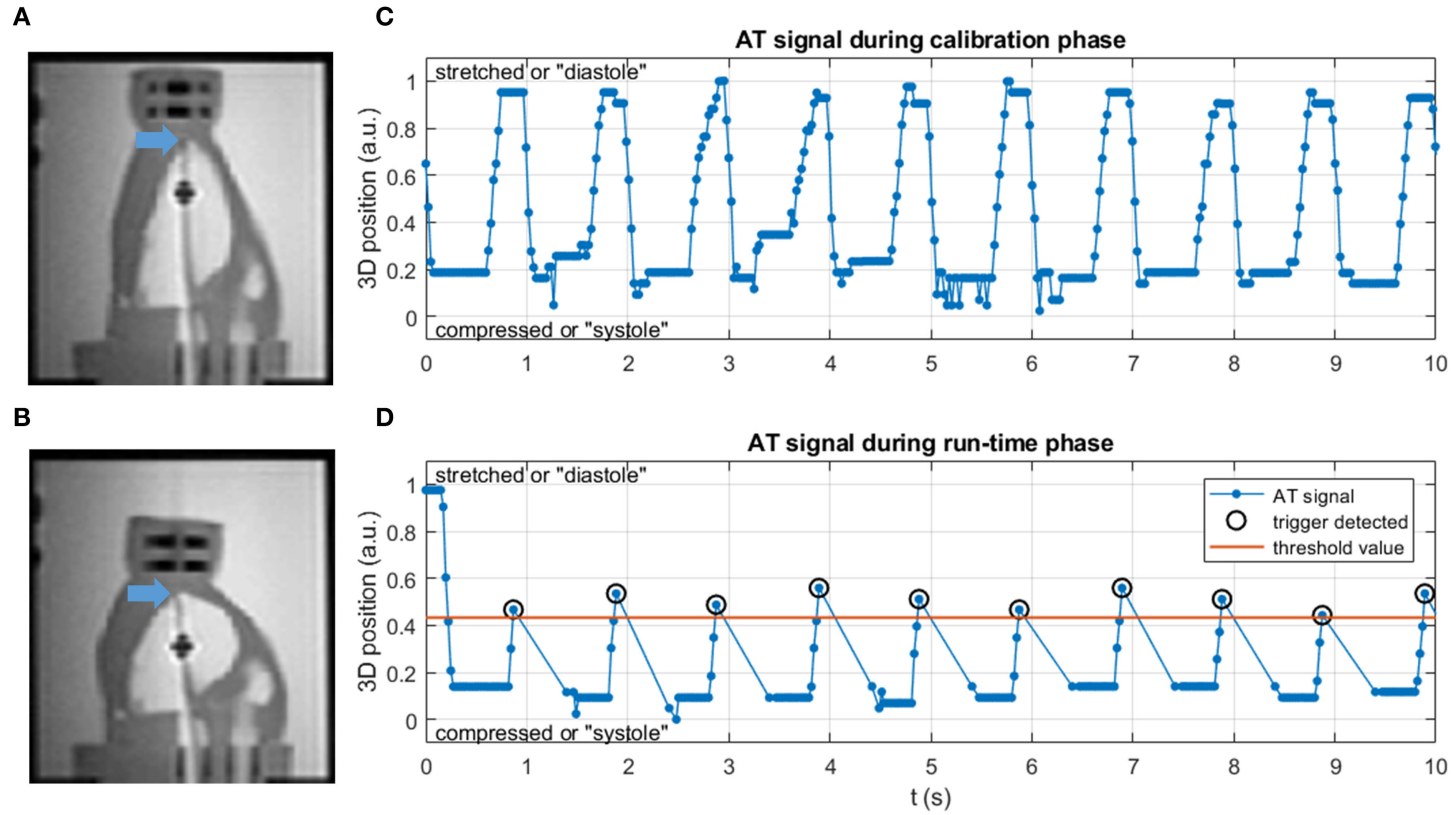
**(A,B)** Coronal MR images of the phantom setup in **(A)** stretched (diastole) and **(B)** compressed (systole) state. Blue arrows mark the catheter tip at the apex of the LV. **(C)** AT signal in the calibration phase is uninterrupted and with a periodicity of 60 BPM. **(D)** AT signal in the run-time phase, with triggers at appropriate motion states and at regular intervals. After each trigger an absence of AT signal is seen, during which the thermometry images were acquired following a trigger delay.

MR images and AT signal of the phantom experiment are shown in Figure 2. The periodic AT signal during the calibration phase (Figure 2C) shows that the precision of the AT location determination was high enough with respect to the cardiac motion to serve as basis for cardiac triggering. Correctly identified triggers and subsequent gaps in the AT signal during run time (Figure 2D) signify that the trigger feedback on the scanner was fast enough for cardiac triggering.

**Figure 3:**
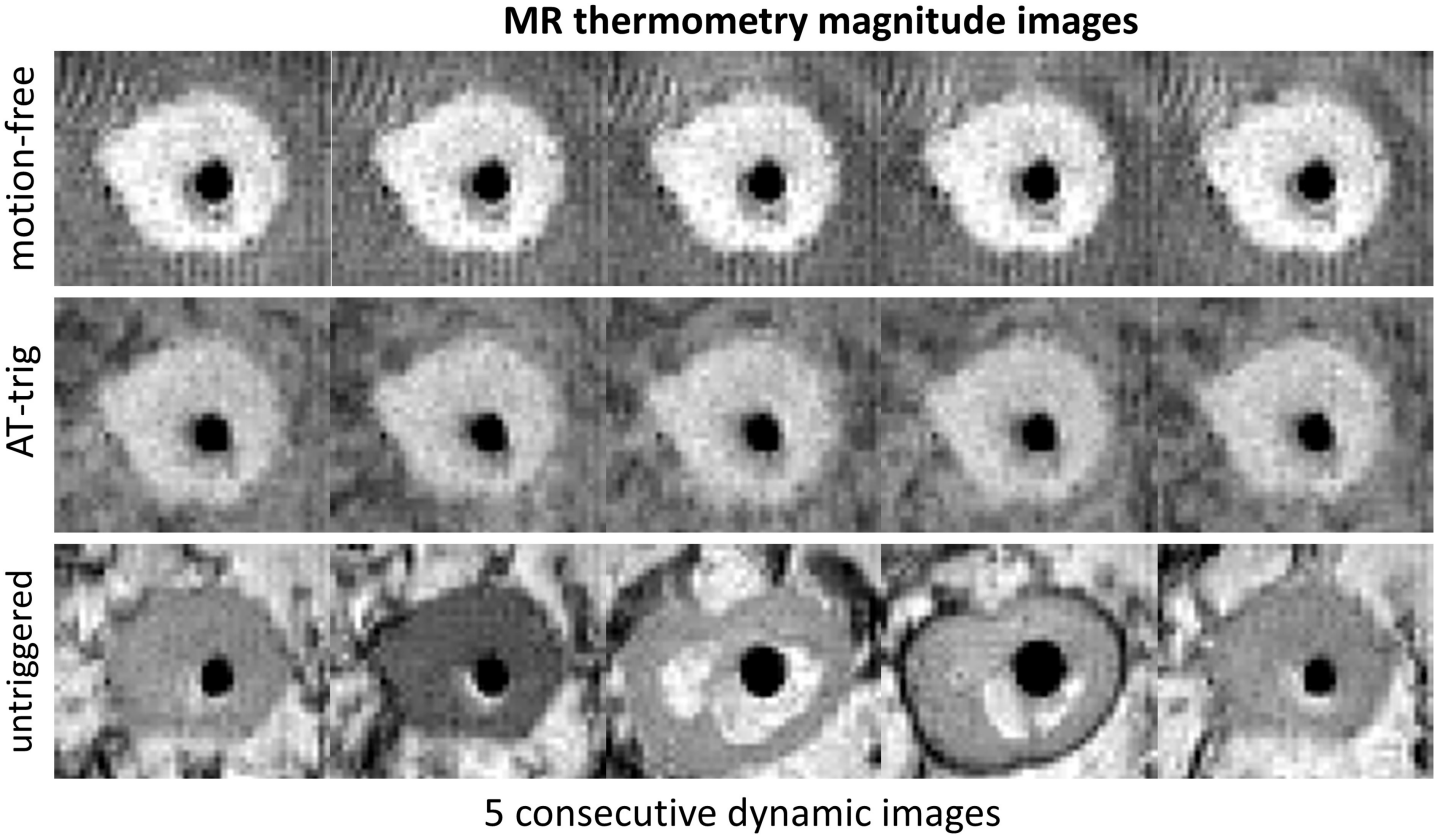
Magnitude images of MR thermometry acquisitions of an apical axial slice of the phantom during the motion-free, AT-trig, and untriggered experiments. The phantom material can be seen as a roughly circular shape with higher signal intensity compared to the darker signal of the water surrounding it. The signal void in the center of the phantom is the piston that drives it into motion. The phantom shape in the images acquired with AT-trig matches the motion-free images, indicating successful triggering. Without triggering, the phantom shape varies under the influence of motion.

Figure 3 shows that magnitude images acquired with AT-trig were all at a consistent cardiac phase, comparable to those acquired in the motion-free experiment. In contrary, the untriggered images were acquired at different cardiac phases. These findings were supported by the temperature stability inside the myocardium-mimicking phantom material: 0.4 ± 0.1 vs. 1.2 ± 0.4 vs. 5.5 ± 3.7 °C for the motion-free, AT-trig, and untriggered experiments, respectively.

### *In vivo* MR–thermometry stability study

**Figure 4:**
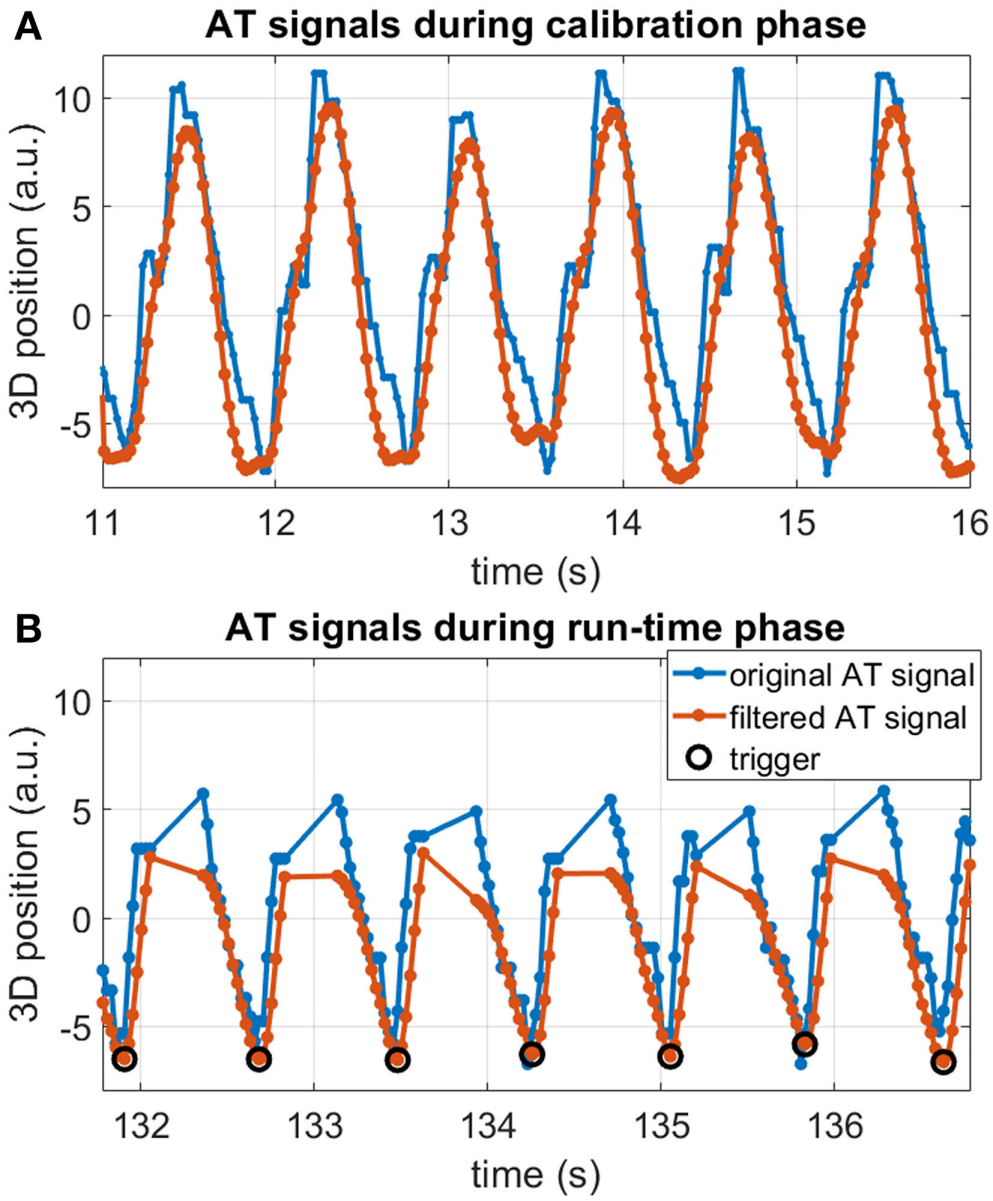
Example of *in vivo* AT signal measured in position #5 in the LV for the valley triggering experiment. Original AT signal (blue), filtered AT signal (orange), and detected triggers (black circles). **(A)** Continuous AT signal acquired during the calibration phase. **(B)** AT signal segment during the run-time phase, showing triggers and AT signal interruptions by imaging blocks. Regular and consistent valley trigger detection was achieved in all cardiac cycles.

After confirming technical feasibility in a phantom, AT signal was successfully measured *in vivo*. This is exemplified in Figure 4, where original and filtered signals are shown in both calibration and run-time phases for one LV location experiment. Although little respiratory motion can be seen in the unfiltered AT signal, the high-frequency components were successfully removed after signal filtering, leading to a smooth triggering signal (Figure 4A). Triggering appears to be successful in the AT signal acquired during run time (Figure 4B).

**Figure 5:**
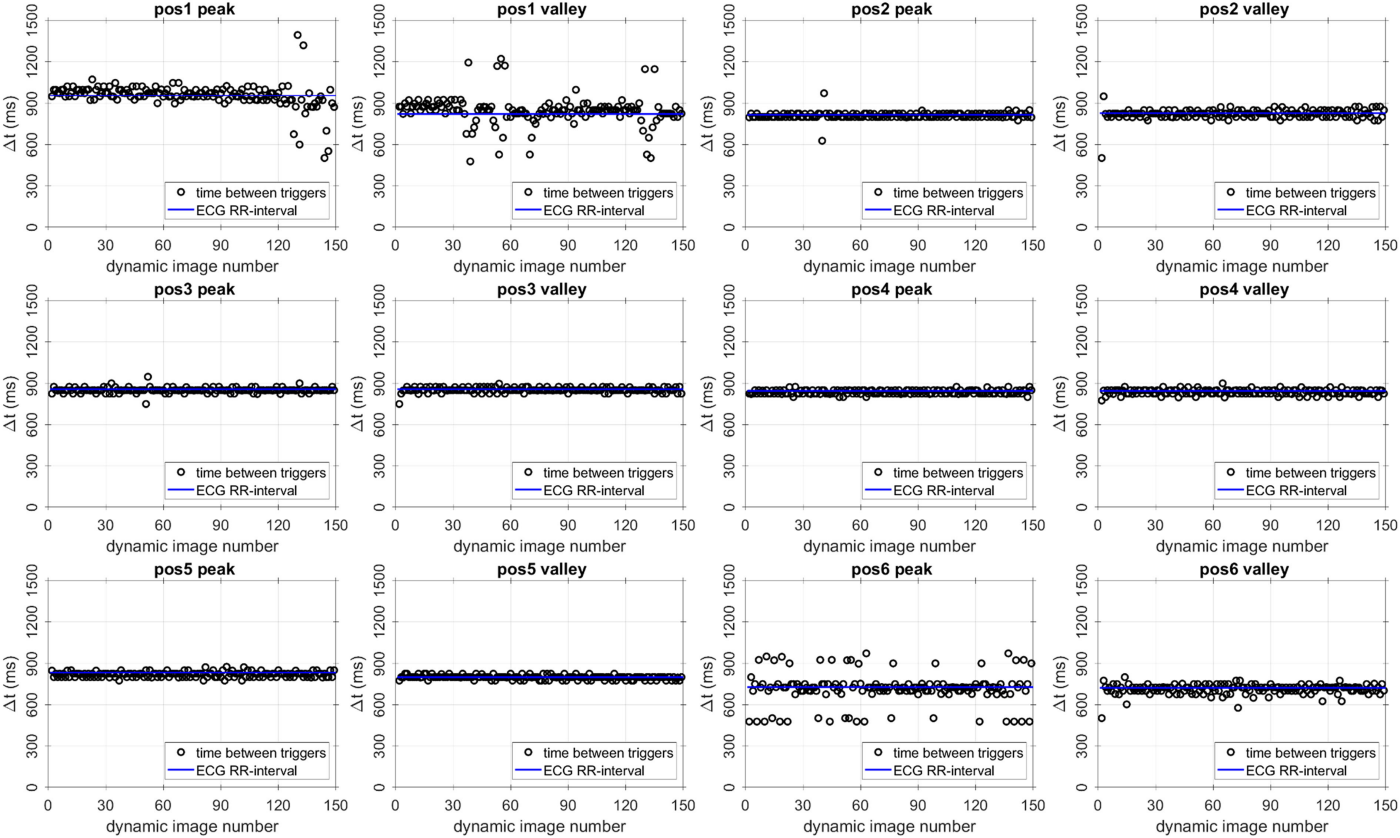
Intervals between triggering events (black circles) and ECG-derived RR interval (blue lines) for all AT-triggered thermometry stability experiments. The AT-trig-derived cardiac cycle length matches the ECG-derived value well throughout the experiments. Pos, position within the LV.

**Figure 6:**
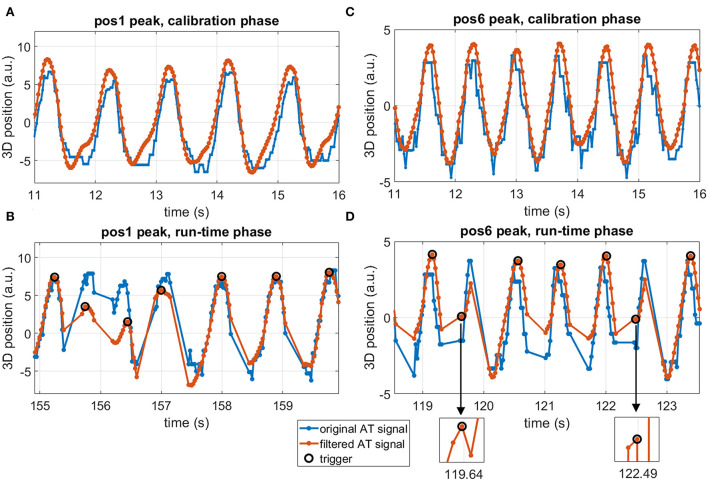
Active tracking signal segments in two experiments with occasional irregular triggers. **(A,B)** position #1 with triggering on peak, calibration phase **(A)** and run-time phase **(B)**. **(C,D)** position #6 with triggering on peak, calibration phase **(C)** and run-time phase **(D)**. Blue lines: original AT signal, orange lines: filtered AT signal, black circles: detected triggers, pos: position within the LV, a.u.: arbitrary units. Irregular triggers around 156 s in **(B)** correspond to actual signal peaks in both unfiltered and filtered signals, suggesting true physiological variations in heart rhythm. Irregular triggers around 119.5 and 122.5 s in **(D)** correspond to local maxima in the filtered signal, as seen in the zoomed inserts, suggesting an improper trigger description for these cardiac cycles.

The intervals between the detected triggers are shown for all 12 AT-trig MR thermometry stability experiments in Figure 5. Minimal variation in trigger intervals was observed for the majority of the cases and corresponded well with the ECG-derived RR interval duration. In three cases (position 1: AT-trig on peak and valley, position 6: AT-trig on peak), a small number of inconsistent trigger intervals were observed suggesting incorrect triggering or irregular heartbeats. Figures 6A,B suggest that the inconsistencies in position 1 were due to actual heart rate variations/arrhythmias since all detected triggers corresponded to real AT signal peak and clear RR variations were observed in the raw AT signal. However, Figures 6C,D show that local maxima in the AT signal in position 6 led to incorrect peak triggering. The mis-triggering rate in position 6 with peak triggering was 13%, with every mis-trigger leading to a pair of shorter and a longer trigger intervals (where the second longer interval compensated for the previous shorter trigger interval).

**Figure 7:**
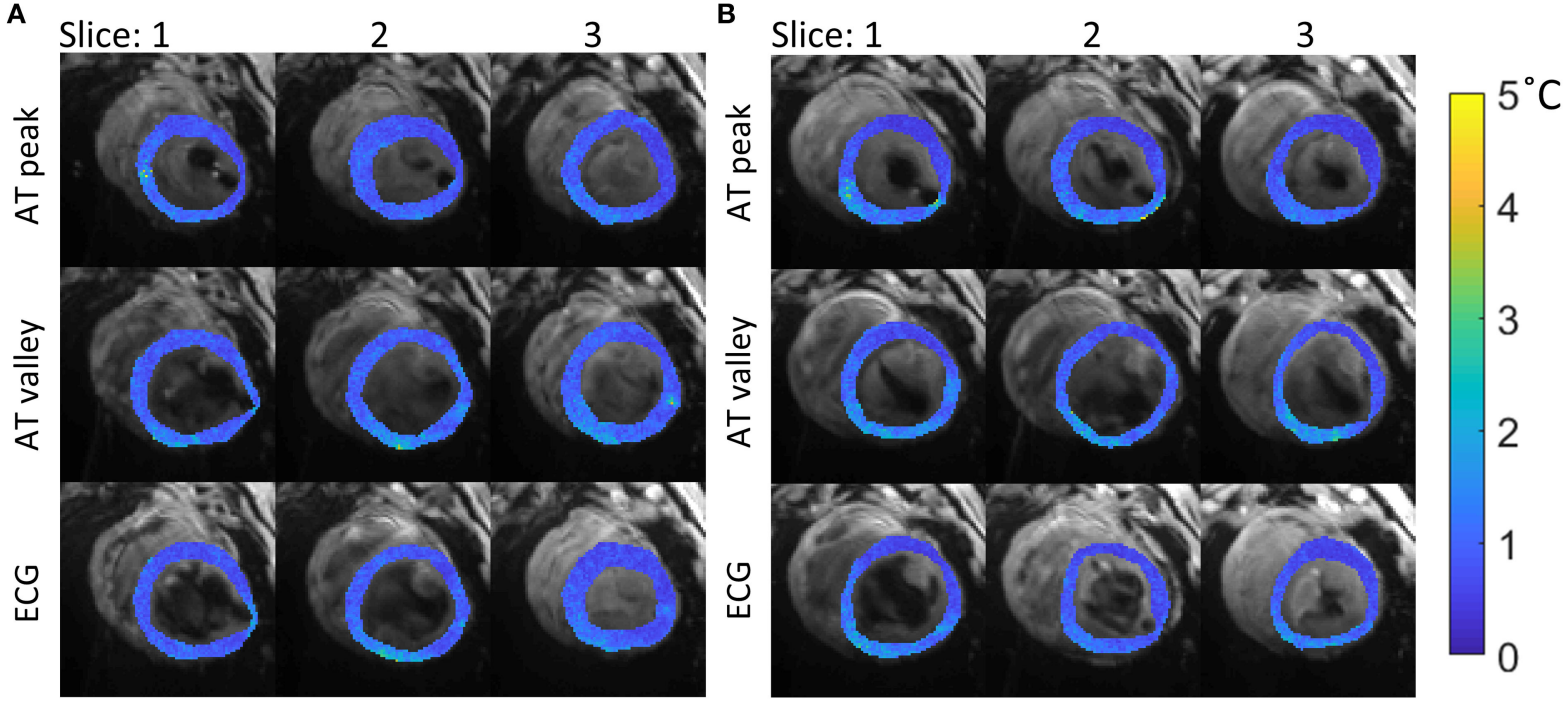
Temperature stability maps of myocardial tissue acquired with the 3 different strategies for cardiac triggering. The stability values (colored maps) are overlaid on average magnitude images (gray scale). Data acquired in 2 different positions in the LV are shown: **(A)** position 4, mean ± sd per method: 0.9 ± 0.3, 1.0 ± 0.4°C and 0.9 ± 0.3°C, for AT peak, AT valley and ECG, respectively. **(B)** position 5 with mean±sd per method: 0.8 ± 0.4, 1.1 ± 0.4, and 0.9 ± 0.4°C, for AT peak, AT valley and ECG, respectively. The mean ± sd per method for all experiments is summarized in Figure 8.

Dynamic series of thermometry images were acquired successfully, and examples of stability maps acquired in 2 different LV positions are shown in Figure 7. Temperature stability obtained with both AT-trig methods (triggering on peak and valley) was comparable to thermometry acquired with ECG triggering, which also performed well in these experiments without RF ablation.

**Figure 8:**
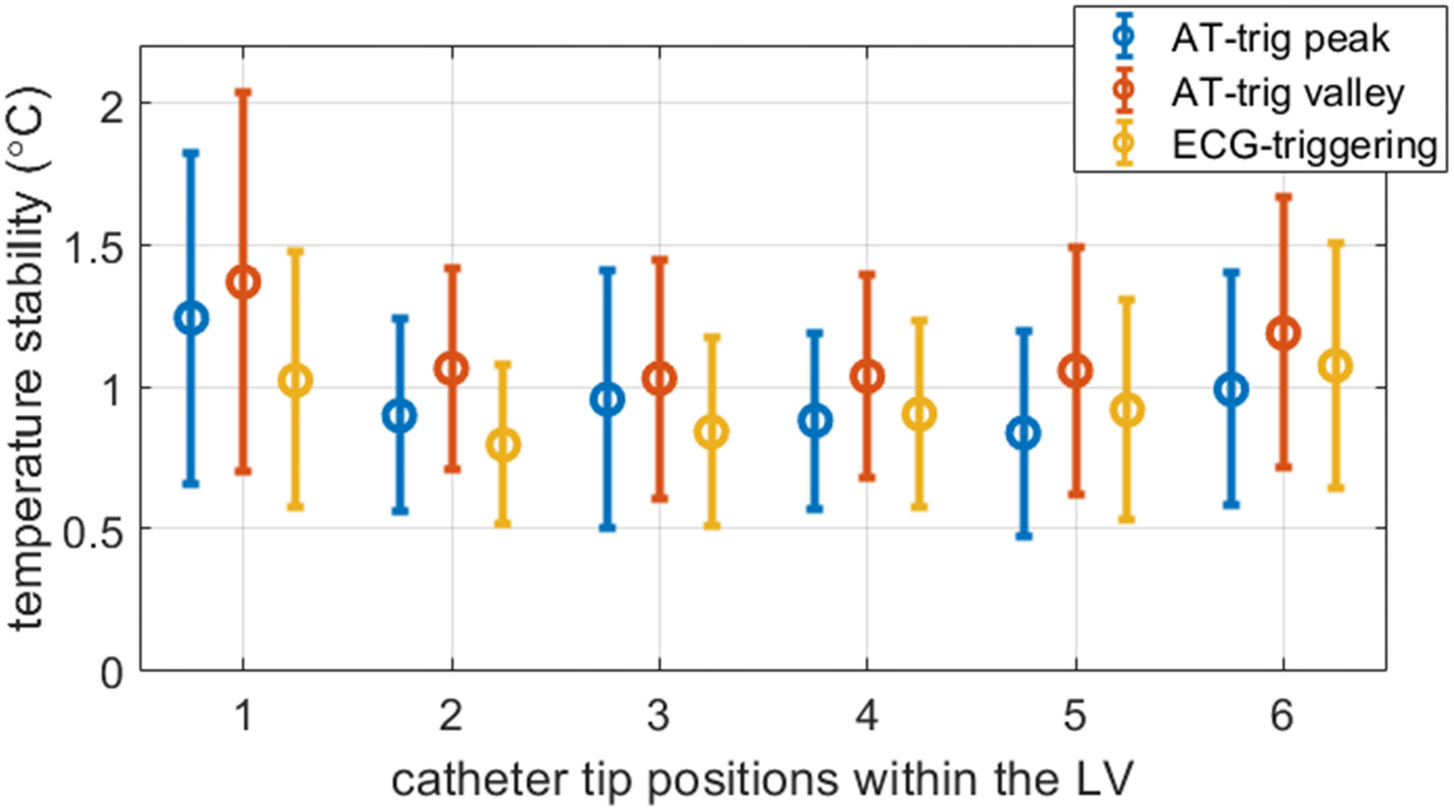
Measured temperature stability in all experiments. Per triggering method, the mean and standard deviation were calculated for all voxels inside myocardial masks combined over the three slices. The 2 AT-trig strategies (AT peak and AT valley) resulted in similar temporal stability compared to ECG-triggered sequences. The precision of scans triggered on the peak of the AT signal matched more closely to ECG-triggered data than scans triggered on the valley of the AT signal. In the first LV position visited in these experiments, the mean and spread in temperature stability measured with both AT strategies were higher than in the other LV positions visited.

Figure 8 shows that similar temperature stabilities were obtained using AT-trig (peak and valley) compared to ECG triggering in each LV position: 1.0 ± 0.4 vs. 1.1 ± 0.5 vs. 0.9 ± 0.4°C, respectively. Overall, triggering on the peak of the AT signal tended to result in slightly better stability compared to triggering on the valleys of the AT signal.

### Feasibility study during *in vivo* ablation

**Figure 9:**
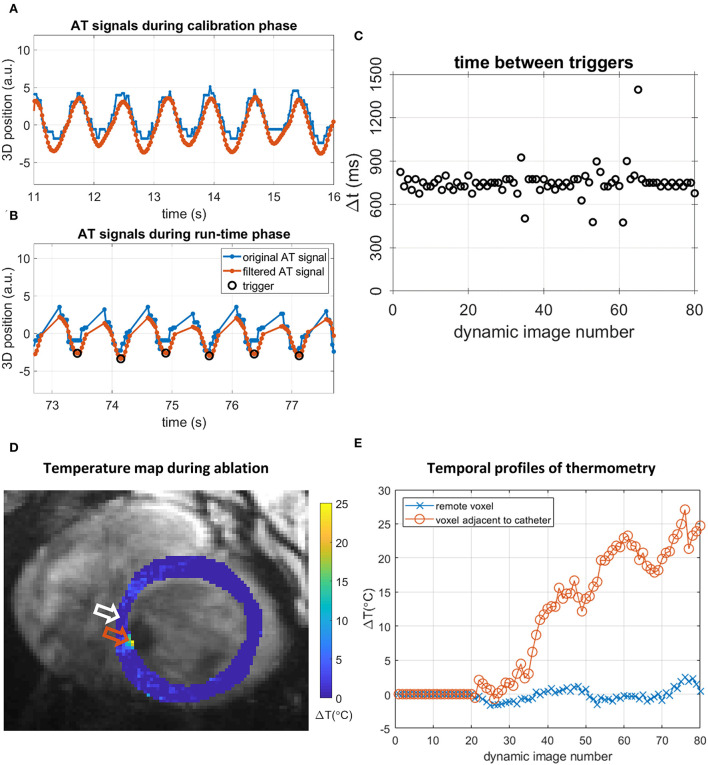
AT-based cardiac triggering and thermometry data during the RF ablation experiment in position #6 in the LV, using AT valley triggering. **(A,B)** AT signals and triggers in 5-s segments. Original AT signal (blue), filtered AT signal (orange), and detected triggers (black circles). **(A)** Continuous AT signal acquired during the calibration phase. **(B)** AT signal segment during the run-time phase, showing triggers and AT signal interruptions by imaging. **(C)** Intervals between triggering events (black circles) during the run-time phase. **(D)** Thermometry map acquired during ablation with AT valley triggering in position 6, 63rd dynamic image. A localized temperature rise is seen near the orange arrow, adjacent to signal void caused by the presence of the catheter in the blood pool. **(E)** Temporal profile of temperature changes in 2 voxels: a voxel remote from the catheter [white arrow in **(D)**] shows temperature changes around 0°C whereas a voxel adjacent to the catheter [orange arrow in **(D)**] displays an elevated temperature during ablation.

Successful AT-triggered MR thermometry during an ablation is shown in Figure 9, where a localized temperature rise can be observed while the majority of the myocardium remains unaffected. AT and thermometry data up to dynamic 80 are shown. More extended data acquisition was limited by the onset of ventricular tachycardia after that point.

## Discussion

AT-based cardiac triggering MR thermometry was successfully demonstrated in a beating heart phantom and *in vivo* in a porcine animal model. *In vivo* trigger intervals recorded with AT-trig closely matched the RR interval derived from ECG, with a low rate of mis-triggers. Temperature stability was comparable to that of the standard ECG triggering in all *in vivo* experiments. Finally, an example of MR thermometry acquired using AT-trig was shown during *in vivo* RF ablation.

*In vivo* cardiac triggering was successful in most AT-trig acquisitions, with persistent mis-triggers observed in only 1 out of 12 AT-triggered thermometry stability experiments, in 13% of the images of this experiment. These mis-triggers were due to local maxima in the AT signal; the use of an improved AT signal filter and/or higher *S*_*Thres*_ value for the definition of cardiac triggers is likely to enable more robust triggering.

Active tracking signal peaks can represent different cardiac motion states (diastole or systole) depending on the orientation of the heart and the catheter inside it. Therefore, some of the 6 locations acquired with peak triggering used a systole-based trigger while others used a diastole-based trigger, which could have had an impact on the stability of MR thermometry and may explain the differences observed between peak and valley triggering. The trigger delay was fixed in these experiments aimed at showing the feasibility of AT-trig. Optimization of the trigger delay for each position within the LV should result in more consistently imaging the same cardiac phase. In the future, an AT-triggered CINE scan could be developed to facilitate the selection of triggering condition (peak vs. valley) and trigger delay needed for imaging of a desired cardiac phase.

The catheter that was used in this study contained 2 microcoils to determine tip location, while for AT-trig, only one coil was used. Using both AT coils might improve the SNR of the location determination, although this study does not suggest that the SNR was insufficient. The use of 2 microcoils could also be used for outlier detection by comparing the positions of both coils.

The respiratory-induced motion of the heart was small in the employed porcine model, when compared to human. Therefore, the full potential of the respiratory motion filter of the AT signal could not be investigated to its full extent in this study. However, the potential of this filter to demodulate cardiac and respiratory motion components from the catheter motion has been demonstrated in prior studies using recorded catheter displacement from standard electrophysiology procedures and standard RF catheters (22). Therefore, it is expected that the employed filter should translate into similar benefit in the proposed application.

A total of six LV locations in a porcine heart were included in this study of AT-trig. Although these locations were selected to sample different myocardial segments, the performance of the technique over a higher LV sampling remains to be investigated. Further evaluation in patients is also needed to study the performance of AT-trig in a clinical setting. Moreover, only one ablation experiment was performed in this proof-of-concept study. The lesion(s) generated through RF ablation and measured with AT-triggered MR thermometry was not assessed and compared to other (MR) imaging methods. It was shown purely as example. Larger-scale experiments will be needed to validate the potential of AT-trig MR thermometry to predict RF ablation lesions, which was beyond the scope of this study.

The calibration phase is currently performed for each run of the triggered thermometry sequence. The cardiac motion of the heart is spatially varying in amplitude and direction, thus requiring a new calibration at each location using the proposed AT-trig approach. The development of an AT-based cardiac trigger that is invariant to motion amplitude and direction may avoid repeating the calibration step at each location and remains to be investigated.

AT-based cardiac triggering might also be used to trigger other cardiac MR sequences used during the intervention such as post-ablation imaging with a long inversion time to visualize ablation-induced changes in T1 (14). Furthermore, retrospective cardiac synchronization of MR sequences (25, 26) and motion correction (27) using AT have also been suggested.

AT-based cardiac triggering provides a direct measurement of cardiac motion as opposed to conventional ECG. This information could therefore be used for detecting and rejecting arrhythmias based on trigger interval or other deviations in the measured motion pattern. Other events that could hamper the ablation therapy efficacy might also be detected using continuous AT, such as the catheter drifting away from the targeted ablation position or poor catheter contact. Finally, AT can also be used to provide prospective slice tracking and could be easily combined with the proposed sequence (28).

## Conclusion

Continuously acquired AT signal for prospective cardiac triggering of an MR-thermometry sequence was feasible in phantom and *in vivo* experiments. AT-trig MR thermometry led to comparable temperature stability as conventional ECG-triggered thermometry and could serve as alternative strategy in situations where ECG triggering is not effective.

## Data availability statement

The raw data supporting the conclusions of this article will be made available by the authors, without undue reservation.

## Ethics statement

The animal study was reviewed and approved by American Preclinical Services (APS) Institutional Animal Care and Use Committee (IACUC).

## Author contributions

Conception of the study: TS and SR. Design of the study: RMo, RR, RN, and SR. Development of the ATrig technique: RMo, RS, AK, RN, and SR. Data acquisition: RMo, KE, JS, AN, RMu, SW, TL, MO'N, RN, and SR. Draft of the manuscript: RMo and SR. All authors contributed to the manuscript, read, and approved the submitted version.

## Funding

This work was supported by the Wellcome Engineering and Physical Sciences Research Council (EPSRC) Center for Medical Engineering at King's College London (WT 203148/Z/16/Z), the EPSRC grant (EP/R010935/1), the innovate UK grant (68539), and the British Heart Foundation (BHF) grants (PG/19/11/34243 and PG/21/10539), and the Wellcome Trust Health Innovation Challenge Fund grant (HICF-R10-698). This research was also supported by the National Institute for Health Research (NIHR) Biomedical Research Center based at Guy's and St Thomas' National Health Service (NHS) Foundation Trust and King's College London, the NIHR Healthcare Technology Co-operative for Cardiovascular Disease at Guy's, and St Thomas' NHS Foundation Trust. This research was supported by the British Heart Foundation Centre for Research Excellence at the University of Edinburgh (RE/18/5/34216). SW is supported by the British Heart Foundation (FS/20/26/34952). This research was funded in whole, or in part, by the Wellcome Trust (WT203148/Z/16/Z and HICF-R10-698). For the purpose of open access, the author has applied a CC BY public copyright licence to any Author Accepted Manuscript version arising from this submission.

## Conflict of interest

Author RMo was seconded to and RN was employed by Siemens Healthcare Ltd. RS and AK were employed by Siemens Healthcare GmbH. KE, JS, and TL were employed by Imricor Medical Systems. The remaining authors declare that the research was conducted in the absence of any commercial or financial relationships that could be construed as a potential conflict of interest.

## Publisher's note

All claims expressed in this article are solely those of the authors and do not necessarily represent those of their affiliated organizations, or those of the publisher, the editors and the reviewers. Any product that may be evaluated in this article, or claim that may be made by its manufacturer, is not guaranteed or endorsed by the publisher.

## Author disclaimer

The views expressed are those of the authors and not necessarily those of the NHS, the NIHR or the Department of Health and Social Care.
